# Disorders of Copper Metabolism in Children—A Problem too Rarely Recognized

**DOI:** 10.3390/metabo14010038

**Published:** 2024-01-07

**Authors:** Sabina Więcek, Justyna Paprocka

**Affiliations:** 1 Department of Paediatrics, Faculty of Medical Sciences, Medical University of Silesia, 40-055 Katowice, Poland; 2 Department of Paediatric Neurology, Faculty of Medical Sciences, Medical University of Silesia, 40-055 Katowice, Poland

**Keywords:** copper metabolism, Menkes disease, Wilson’s disease, children

## Abstract

Copper plays an important role in metabolic processes. Both deficiency and excess of this element have a negative effect and lead to pathological conditions. Copper is a cofactor of many enzymatic reactions. Its concentration depends on the delivery in the diet, the absorption in enterocytes, transport with the participation of ATP7A/ATP7B protein, and proper excretion. Copper homeostasis disorders lead to serious medical conditions such as Menkes disease (MD) and Wilson’s disease (WD). A mutation in the *ATP7A gene* is the cause of Menkes disease, it prevents the supply of copper ions to enzymes dependent on them, such as dopamine β-hydroxylase and lysyl oxidase. This leads to progressive changes in the central nervous system and disorders of the connective tissue. In turn, Wilson’s disease is an inherited autosomal recessive disease. It is caused by a mutation of the *ATP7B gene* encoding the ATP7B protein which means excess copper cannot be removed from the body, leading to the pathological accumulation of this element in the liver and brain. The clinical picture is dominated by the liver, neurological, and/or psychiatric symptoms. Early inclusion of zinc preparations and chelating drugs significantly improves the prognosis in this group of patients. The aim of the study is to analyse, based on the latest literature, the following factors: the etiopathogenesis, clinical picture, diagnostic tests, treatment, prognosis, and complications of disease entities associated with copper disturbances: Menkes disease and Wilson’s disease. In addition, it is necessary for general practitioners, neurologists, and gastroenterologists to pay attention to these disease entities because they are recognized too late and too rarely, especially in the paediatric population.

## 1. Introduction

Copper is a trace element essential to life. It is the cofactor of many enzymes, including amine oxidase, copper-dependent superoxide dismutase, cytochrome c oxidase, dopamine beta-hydroxylase, and tyrosinase. (Table 1). Copper participates in the reactions of disproportionation, hydroxylation, and oxidation [1,2,3].

In the human body, excess copper may cause the oxidation of proteins and lipids. In pathological conditions. copper can bind directly to the DNA and it can change the structure of chromatin. Copper can generate free radicals because Cu^2+^ can be reduced to Cu^+^, in the presence of reducing agents, like ascorbic acid or reduced glutathione, which have the capacity to catalyse hydroxyl radical formation via Haber–Weiss reaction and it can induce oxidation of bases and DNA strand breaks [1,2,4].

The body of a healthy adult contains approximately 80–100 mg of copper, accumulated mainly in the bones, liver, and muscles. The average daily intake of copper amounts to around 2–4 mg. The food sources of copper include meat, crustaceans, nuts, wholemeal foods, and dried fruit. Around 50% of copper is absorbed in the stomach, duodenum, and the initial parts of the small intestine, and the remaining amount is excreted in faeces. Once absorbed, copper bound to albumins is transported to the liver. In the liver, it is taken up by hepatocytes and partially excreted with bile. The ATP7B protein in the Golgi apparatus in hepatocytes enables the excretion of copper into the bile and is necessary for the intracellular binding of copper with apoceruloplasmin, which after binding 6 atoms of copper is released into the blood as ceruloplasmin [1,2,3].

Ceruloplasmin is an alpha 2 globulin with a molecular weight of approximately 160 kDa. It contains 70–90% of the copper found in plasma. Each molecule of ceruloplasmin strongly binds 6 atoms of copper which makes this element not easily replaceable. The remaining 10% of plasma copper is transported with albumins or forms a complex with free histidine. Additionally, ceruloplasmin is also ferroxidase and promotes Fe^2+^ to Fe^3+^ oxidation. This activity is fundamental for iron incorporation into transferrin and, therefore, for iron homeostasis. Copper excess is excreted via the gastrointestinal tract. Metalothioneins are proteins of a small molecular weight of about 6.5 kDa, present in the cytosol of liver, kidney, and intestinal cells. They contain large amounts of cysteine and are capable of binding copper, zinc, cadmium, and mercury. When administered over a short period of time, e.g., as an injection, copper induces an increase in the amount of those proteins in tissues. These proteins may participate in the storage of the non-toxic form of copper and contribute to the metabolism of the system. The binding of copper and its storage reduces the amount of this element, which participates in the formation of free radicals [1,3,5,6]. The basic laboratory tests assessing the transformations of copper and their normal values are presented in Table 2 [1,3,6,7,8,9] (Table 2).

## 2. Wilson’s Disease (Hepatolenticular Degeneration, OMIM #27790)

The condition is inherited as an autosomal recessive trait.

Wilson’s disease is characterised by the inability to excrete excess copper from the liver with the bile and it leads to its accumulation in the liver, brain, kidneys, and red cells.

The copper binding to apoceruloplasmin in the Golgi apparatus cistern is mediated by the ATP7B protein. Because lack of catalytic activity of ATP7B in Wilson’s patients despite the normal synthesis level of ceruloplasmin this protein is mainly in apo form, which is unstable and degraded.

Over 800 mutations of the gene in Wilson’s disease have been described. In most patients with Wilson’s disease, there are focal nonsense mutations, which are responsible for the structural changes within ATP7B membrane domains. In most patients with Wilson’s disease, two different mutations in the alleles are observed, one mutation in 30% and none in 10% of the patients. No unequivocal correlation between the mutation type and the clinical symptoms, and the severity of the condition has been reported. Among the European population, the most common is the H1069Q mutation. The relationship between genotype and phenotype is unclear even now. Patients with the H069Q mutation in the *ATP7B gene* show phenotypic variations [7,10,11,12].

The prevalence of Wilson’s disease is estimated at 1/30,000 births; however, it seems it may be higher. In the course of the disease, both functions of ATP7B are impaired; hence, the lower concentration levels of ceruloplasmin in the blood, with only unstable apoceruloplasmin secreted into the blood. At the same time, ionised copper is retained in the liver and then in other organs. Copper redistribution may be asymptomatic, but if it happens dramatically, it is accompanied by hepatocyte necrosis and liver failure. In the central nervous system, the highest concentration levels of copper are observed in the basal ganglia [7,10,11,12].

### 2.1. Symptoms of Wilson’s Disease

Symptoms of Wilson’s disease usually occur between 5 and 35 years. Untreated Wilson’s disease leads to death due to liver failure and/or damage to the CNS. In the clinical picture of Wilson’s disease, two phenotypes are highlighted, namely the hepatic and neuropsychiatric ones [7,12,13,14,15,16].

The hepatic manifestations are quite varied and may range from asymptomatic laboratory abnormalities and steatosis to acute hepatitis, acute liver failure, chronic hepatitis, and cirrhosis. Abdominal pain, lack of appetite, loss of body weight, jaundice, ascites, and/or bleeding from the gastrointestinal tract are sometimes reported in the medical interview. Jaundice may be the symptom of increased haemolysis in the course of the disease and/or liver decompensation—it is, however, always an unfavourable prognostic indicator. Acute liver failure is usually associated with Coombs-negative hemolytic anaemia, aspartate aminotransferase to alanine aminotransferase ratio that is often greater than 2, normal or subnormal alkaline phosphatase, coagulopathy unresponsive to vitamin K, encephalopathy and renal failure [7,12,13,14,15].

The neurologic manifestations, predominantly extrapyramidal, may develop insidiously or precipitously and the severity of symptoms often fluctuates, sometimes during the same day. Neurologic abnormalities are more common in adults, but they can be observed in early childhood. Dysarthia (difficulty with speech) is the most common first manifestation. The other symptoms include bradykinesia, facial grimacing, tremor, dystonia, rigidity, urinary incontinence, and hyperreflexia. The psychiatric symptoms sometimes precede the onset of the neurological ones and mostly involve depression, acute personality changes, aggressiveness, and irritability [7,10,14,15,16]. Clinical symptoms are shown in Table 3.

Wilson’s disease should be considered in children above the age of 1; however, it rarely occurs below the age of 10 [7,12,14,15,16,17].

Ocular manifestations and Kayser–Fleischer rings are the classic ocular features. Kayser–Fleischer rings are seen as a grey-brown opacity in the peripheral cornea (deposits of copper-rich and sulphur-rich granules in the Descemet membrane). It first develops superiorly, then inferiorly, and, finally, in the lateral and medial areas of the cornea. These occur in approximately 40% of patients with hepatic manifestations and in about 95% of patients with neurologic manifestations [8,17,18].

### 2.2. Diagnostics of Wilson’s Disease

Both the clinical picture and laboratory test results are not specific to Wilson’s disease. The most important tests in the diagnostics of Wilson’s disease involve the concentration levels of ceruloplasmin in the blood plasma, 24 h urinary copper excretion, and molecular tests [10,15,17,18,19,20,21,22].

- Concentration levels of ceruloplasmin in blood plasma are reduced in 80–95% of patients; however, it needs to be pointed out that lower concentration levels of ceruloplasmin may be the result of every advanced stage of liver disease impairing the synthesis of protein. Lower concentration levels of ceruloplasmin are also observed in malnutrition, enteropathies with protein loss, Menkes disease, nephrotic syndrome and inherited aceruloplasminemia. However, normal ceruloplasmin concentration levels do not eliminate Wilson’s disease.
- Urinary copper excretion is increased and only concerns non-ceruloplasmin-bound copper. This test is greatly significant in patients with suspected Wilson’s disease and should be conducted routinely. In diagnostically doubtful cases, penicillamine-induced (single dose of 500 mg) renal copper excretion may be used.
- Plasma copper concentration in those patients is usually lower, which is due to the reduced concentration level of ceruloplasmin.
- The histopathological examination of the liver specimen shows signs of steatosis, fibrosis with inflammation, and/or cirrhosis. Additionally, rhodamine stain can be used to detect copper deposits in the liver. A hepatic copper concentration greater than 250 ug/g dry weight in the absence of cholestasis is diagnostic of Wilson’s disease.
- A molecular test is done to confirm the diagnosis. A negative result, however, does not exclude Wilson’s disease, as new mutations within the ATP7B protein coding gene have been detected. Also, the condition may be related to microdeletions.
- In patients with neurological and/or psychiatric symptoms, the MRI examination shows increased intensity of the basal ganglia in the T2 sequence [3,18,19].

Wilson’s disease scoring system (by Peter Ferenci) is also useful in the diagnostic process. A minimum score confirming the diagnosis is 4 points (Table 4) [20,21].

### 2.3. Differential Diagnostics

Differential diagnostics of Wilson’s disease include:

- Autoimmune hepatitis;
- Non-alcoholic fatty liver disease;
- Viral hepatitis;
- Deficiency in alpha-1-antitrypsin;
- Metabolic diseases: hemochromatosis;
- Post-drug reactions;
- Nieman–Pick C type disease;
- Congenital disorders of glycosylation;
- Aceruloplasminemia [1,3,6,7,9,15,23].

### 2.4. Treatment of Wilson’s Disease

The aim of the treatment is the inhibition of the progression of the disease and the regression of the lesions in the liver, CNS, and other organs. The medication used in the treatment of Wilson’s disease can be divided into the following:

1. Preparations facilitating renal copper excretion which have been bound in the bloodstream via the chelating mechanism—penicillamine, trientine, and tetrathiomolybdate.
2. Medication inhibiting intestinal copper absorption (zinc sulphates) [1,7,18,22,23,24,25,26,27,28,29,30].

Treatment of Wilson’s disease is presented in Table 5.

The bioavailability of copper, so the percentage of ingested copper that can be absorbed, changes under the influence of zinc, which reduces copper absorption. The treatment of Wilson’s disease requires the monitoring of urinary copper excretion and zinc levels—if zinc-based therapy is applied [18,22,23,24,25,26,27,28].

Bis-choline tetrathiomolybdate (Decuprate) is a new drug for the treatment of Wilson disease presenting with acute neurological disease as conventional chelator therapy could prove unsuccessful in such patients, and lead to rapid and irreversible clinical deterioration.

Exciting research progress is being made in the development of curative strategies for Wilson's disease. They include gene therapy, cell therapy, and correction of dysfunctional ATP7B mutant function [18,23].

Additionally, it is recommended to avoid foods high in copper—crustaceans, nuts, dried fruit, chocolate, cocoa, mushrooms, and liver [31].

In acute liver failure with hepatic encephalopathy, liver transplant remains the first-line treatment. Plasma exchange should be considered as a treatment for fulminant Wilson’s disease in children and young adults, at least as a bridge to transplantation. Acute liver failure without encephalopathy may in many cases be successfully treated pharmacologically using chelators; however, some patients need a liver transplant. Neurological symptoms are not an indicator of liver transplant. Table 6 shows the prognostic scoring system for the assessment of the indicators for liver transplant in acute liver failure in the course of Wilson’s disease. A total score of 11 and more yields an unfavourable prognostic without a liver transplant [6,7,14,29,30,31,32].

Another approach is based on the replacement of healthy hepatocytes using cell therapy to restore physiological ATP7B-dependent copper excretion into bile fluid. The dysfunctional liver tissue needs to be repopulated with healthy liver cells that are then able to proliferate into functional hepatocytes and reconstitute the biliary canalicular network [14,30,32,33].

## 3. Menkes Disease (Kinky Hair Disease, Trichopoliodystrophy, Steely Disease—OMIM#309400)

Menkes disease is caused by the mutations in the gene *ATP7A* responsible for the synthesis of the copper-binding ATP7A protein. The disease is correlated with chromosome X and mainly concerns male infants. More than 350 Menkes disease-causing mutations are described and represented by small deletions/insertions, nonsense mutations, missense mutations, and splice sites in roughly equal proportions [1]. About 1/3 of cases of Menkes disease arise from de novo mutations. The prevalence of the disease is estimated at 1:140,000 males [34,35]. Menkes disease was first described in 1962 by Menkes et al. who reported the affected subjects, all of them male and from the same family [31]. In 1993, it was discovered that the disease is caused by a mutation in the gene encoding copper-binding P-type ATPase—*ATP7A gene*/ATP7A protein. It is believed that the ATPase is responsible for the excretion of copper from cells, including enterocytes into the bloodstream, and a lack of ATPase is the cause of systemic copper deficiency. In patients, copper can be seen accumulating in enterocytes and renal tubules. Low levels of brain copper in Menkes disease are likely associated with the deficient function of several copper-containing enzymes in the nervous system.

Lack of or decreasing activity of copper-dependent enzymes such as cytochrome C oxidase, superoxide dismutase, tyrosinase, and lysyl oxidase can lead to disturbances in the functioning of the nervous systems (Table 7) [2,36,37,38,39,40,41].

Additionally, the role of copper as a modulator of hippocampal synaptic transmission has been suggested [37,41,42,43].

### 3.1. Symptoms of Menkes Disease

The pathological changes in Menkes disease affect the nervous system, the connective tissue, and vessels. Symptoms of the disease can be observed since infancy. The pregnancy is usually uncomplicated. There may be premature labour and delivery, but most male patients are born at term with appropriate birth measurements. In the early neonatal period, patients may present with prolonged jaundice, hypothermia, hypoglycaemia, and feeding difficulties. The first sign of Menkes disease may be unusually sparse and lustreless scalp hair that becomes tangled on the top of the head at the age of 1–2 months.

The patients are typically diagnosed at 3–6 months of age, often due to the association of seizures with abnormal hair, which is the striking feature of the disease. The symptoms increase gradually with age and lead to death usually before the age of 4 years of the life. In the standard form of the disease, there is a complete lack of activity of the ATP7A. The clinical picture is dominated by regression in the development of psychomotor activity, reduced muscle power and muscle tension, visual disturbances, and seizures [43,44,45,46]. The following phenotypic traits are noticeable during the physical examination: short, curly, coarse, and steel wool hair, often hypopigmented, cherubic face—wide cheeks, reduced facial movements, depressed nasal bridge, and ptosis. Neurological manifestations are likely related to the perturbations in the copper-dependent enzymatic pathways involved in neurotransmitter and energy metabolism. Morphological changes suggest diffuse cerebral and cerebellar atrophy with loss of volume of both grey and white matter [33,36,42,45]. Neuropathological findings in the nervous system in Menkes disease are presented in Table 8.

The literature usually mentions focal seizures with progression to myoclonia in terms of chronic late-stage epilepsy. Tonic seizures and myoclonic jerks with multifocal epileptiform activity on the EEG are also reported. Focal status epilepticus is characteristic in the early stage of the disease, followed by infantile spasms and multifocal seizures. Typical EEG changes include ictal rhythms, focal, unilateral or bilateral, over posterior regions, while interictal changes include polymorphic slow, spike and waves, and multifocal epileptiform abnormalities in the first stage. Morphological correlates with changes in the brain (atrophy of grey matter, ventriculomegaly, tortuous intracranial vasculature, and white matter signal changes consistent with loss of myelin and axons). The history of epilepsy is usually characterized by 3 stages:

1. Focal clonic seizures. Status epilepticus occurring at mean 3 months of age;
2. Intermediate-infantile spasms;
3. Late-multifocal, myoclonic, and tonic seizures [33,34,36,42].

Muscle tone is often decreased in early life, but is later replaced by spasticity and weakness of the extremities. Micrognathia and an ogival palate can occur. There is widespread cerebral and cerebellar degeneration, tortuosity of blood vessels, bladder diverticula, and skeletal abnormalities. Radiologically, osteoporosis of the long bones, metaphyseal spurs, and numerous wormian bones near the lambdoid sutures are typical findings. Aneurysms can occur, leading to subdural, cerebral, or intestinal haemorrhages. Also, difficulties swallowing and feeding, increased vomiting and recurrent diarrhoea often lead to malnutrition. Late manifestations of this disease are blindness, subdural haematoma, and respiratory failure. Patients with Menkes disease often die due to infections, rupture of the arteries and bleeding into CNS, failure to thrive, and/or respiratory failure [30,32,33,37,40,41,42,45,46].

Allelic variant ATP7A-related distal motor neuropathy (OMIM#277900).

Partial deficiency happens in clinically milder cases when delayed development of psychomotor activity is observed but the patients are capable of walking and talking independently. The most common symptoms include weakened muscle power, muscle tremors, ataxia, and dystonia. Changes in the connective tissue are more common than in the classic form of the disease [35,36,42,46].

The mildest form of Menkes disease is called occipital horn syndrome(OMIM#304150—X-linked cutis laxa).

It involves abnormalities in the connective tissue in teenagers and adults. These abnormalities are related to the excessive elasticity of joints and the skin and the occurrence of bladder and gastrointestinal tract diverticula, and abdominal hernia. Calcifications are a characteristic trait. They are known as occipital horns and cover the area from the trapezius and the sternocleidomastoid muscle to the occipital bone, and can be felt during the physical examination. The first signs can be intractable diarrhoea or urinary tract infections. In spite of these problems, diagnosis of OHS is usually made only around 5–10 years of age. Motor development is delayed due to muscular hypotonia and is associated with unusual clumsiness. Height is usually normal, but with mild disproportion in the length of the trunk, the narrowness of the chest, and pectus deformity. Bilateral inguinal hernias in Menkes disease had previously been described as mild forms of the disease. A particular problem is orthostatic hypotension. The intellectual capacity is described as low to borderline normal [31,35,36,37,41,42,46].

### 3.2. The Diagnostics of Menkes Disease

The diagnostics of Menkes disease involves:

1. The clinical picture.
2. Results of the laboratory tests—the reduction in the concentration levels of copper and ceruloplasmin in blood serum. Serum levels of copper and ceruloplasmin should be measured after the third week, as they can be low in normal children during this time window.
3. Molecular tests—the definite diagnosis of Menkes disease requires the detection of mutations in the *ATP7A gene*; however, a negative result does not eliminate Menkes disease.

Neuroimaging studies frequently disclose cerebral atrophy, areas of low density within the cortex, impaired myelination, and tortuous and enlarged intracranial vessels [1,5,46,47].

The prevalence of congenital heart disease associated with Menkes disease and its variants has not been estimated, but it seems that newborn infants with this disease should be evaluated for heart disease by careful clinical examination and echocardiography (it can be connected with deficiency of lysyl oxidase) [48].

### 3.3. Treatment of Menkes Disease

The administration of copper in the form of histidine complexes improves its absorption and sometimes alleviates the symptoms of the disease. There are studies mentioning the administration of cupric chloride subcutaneously, initially of 250 ug b.i.d, and then of 250 ug q.d. The initial impression was that the treatment response was variable, leaving investigators with the conclusion that early initiation of copper replacement therapy was effective in some individuals. In cases where the deficiency is severe, treatment has been ineffective, as there is insufficient functional protein to carry copper penetrates the blood–brain barrier. The relationship between genotype and the amount of functional protein activity, and the early detection of the disorder in the pre-symptomatic stage is critical in predicting response to therapy. Copper–histidine supplementation is an effective treatment when administered soon after birth, and neurologic development can be maintained. Children with Menkes disease without treatment usually die before the third year after birth due to central nervous system dysfunction [5,33,47].

Cases of female patients with classical Menkes disease are extremely rare, but have been reported. Menkes disease phenotypes have been reported in females with X chromosome; autosome translocations—disrupting *ATP7A gene* function—or *ATP7A gene* alterations. Those females manifest variable clinical findings as pili torti, seizure presence and/or the age of onset, cerebrovascular tortuosity, and degree of intellectual disability. No females with classic Menkes disease have been reported [49,50].

## 4. Aceruloplasminemia

It is a genetic disease characterised by very low concentration levels of ceruloplasmin with a subsequent deficiency in the activity of iron oxidase. This leads to abnormalities in the cellular iron efflux, which then accumulates in some organs such as the CNS, hepatocytes, and pancreatic islets. Patients present with neurological symptoms and diabetes. The treatment involves chelating agents and plasma/ceruloplasmin transfusion [1].

## 5. Copper Deficiency

Copper deficiency is rarely seen. It mainly concerns patients fed parenterally over a prolonged period of time. The clinical picture is dominated by anaemia, leukopenia, hypercholesterolemia, haemorrhagic manifestations, bone demineralisation, and neurological symptoms. Rarer symptoms include increased susceptibility to infections, reduced hair pigmentation, and delayed growth. Cardiological and immunological abnormalities are observed in infants [1].

The comparison of Menkes and Wilson’s disease is presented in Table 9.

## 6. Conclusions

- It is necessary for general practitioners, neurologists, and gastroenterologists to pay attention to diseases connected with copper metabolism because they are recognized too late and too rarely, especially in the paediatric population;
- Patients with serum levels of ceruloplasmins below 120 mg/L and children with urinary copper excretion above 40 ug should undergo genetic testing for Wilson’s disease;
- Only early detection of Menkes and Wilson’s disease will enable a prompt introduction of treatment and reduce the probability of delayed complications.

## Figures and Tables

**Table 1:** Copper requiring enzymes.

Copper Requiring Enzymes	Enzyme Function
Cytochrome c oxidase	Transports electrons from c cytochrome to oxygen in the chain of electron transport
Amine oxidase	Metabolism of neurotransmitters-noradrenaline, dopamine, serotonine Metabolism of amines–histamine, putrescine
dopamineβ-hydroxylase	Hydroxylates dopamine to noradrenaline
Peptidylglycine monooxygenase	Peptin hormone maturation—amidation of alpha-terminal carboxylic acid group of glycine
Ferroxidase (ceruloplasmine, hephaestin)	Oxidises iron activity
Lysyl oxidase	Forms cross-linkages in collagen and elastin
Tyrosinase	Synthesises melanin
Superoxide dismutase	Converts hydrogen superoxide to hydrogen peroxide
Monophenol monooxygenase	Melanin synthesis conversion of tyrosine to DOPA
Methionine synthase	Transfer of methyl group from methyltetrahydrofolate to homocysteine to generate methionine
Adenosylhomocysteine hydrolase	Regeneration of homocysteine from adenosylhomocysteine in the methylation cycle

**Table 2:** Laboratory tests assessing the transformations of copper and their normal values.

Test	Normal Values
Plasma copper level	10–20 umol/L
Concentration of ceruloplasmin in serum	16–60 mg/dL
Urinary copper excretion	<100 mg/24 h
Liver copper level	20–50 ug/g dry weight

**Table 3:** Clinical symptoms of Wilson’s disease.

Organ	Clinical Symptoms
Central Nervous System Neurological and/or psychiatric symptoms 35% patients	Intention tremor Dystonia Ataxia Disorders of posture, balance, and gait Speech disorders Facial tics, dystonic tongue movements Facial amimia Learning difficulties, attention, and concentration disorders Dysphagia Behavioural changes—irritability, aggression, and loss of sexual inhibitions (20%) Dementia Migraines Insomnia Depression Myopathy
Liver 40% patients	Hypertransaminasemia Fatty liver Acute hepatitis Hepatomegaly Cholestasis Acute liver failure (5%) Chronic hepatitis Cirrhosis. Portal hypertension. Liver failure.
Eyes	Kayser–Fleischer ring (green or golden rings encircling the cornea of the eye—a sign of copper deposition in the Descemet’s membrane)—rare in paediatric patients. Sunflower cataract
Red blood cells	Coombs-negative haemolytic anaemia
Kidneys	Defect of the proximal tubule-type 2 tubular acidosis—Fanconi syndrome Nephrocalcinosis
Osteoarticular system	Arthritis-damage to the cartilage Rickets Osteoporosis/Osteopenia
Cardiovascular system	Cardiomyopathy Circulatory insufficiency Cardiac arrhythmia
Endocrine system	Infertility Irregular periods Recurrent miscarriages Damage to the adrenal glands with skin hyperpigmentation Damage to the parathyroid glands with hypocalcaemia Hypothyroidism Gigantism
Other	Weakness, fatigue, loss of appetite Abdominal pain Pancreatitis Loss of body weight Nose bleed Anaemia

**Table 4:** Wilson’s disease scoring system (by Peter Ferenci).

Criterium	Score
Serum ceruloplasmin concentration levels	
>20 mg/L—normal	0
0.1–0.2 g/L	1
<0.1 g/L	2
24 h urinary copper excretion	
<100 mg/24 h—normal	0
1–2× the upper limit of normal	1
>2× the upper limit of normal	2
Normal but >5× after penicillamine	2
Copper levels in the liver tissue	
<50 u/g dry weight	−1
<5× the upper limit of normal (50–250 u/g dry weight)	1
>5× the upper limit of normal (>250 u/g dry weight)	2
Rhodamine stain (when the copper content in the liver tissue has not been marked)	
None	0
Present	1
Mutations	
Two mutations	4
One molecular variant	1
No mutations	0
The Kayser–Fleischer ring	
Present	2
Absent	0
Neurological symptoms	
Advanced	2
Mild	1
None	0
Coombs-negative anaemia	
Present	1
Absent	0

**Table 5:** Treatment of Wilson’s disease.

Medication	Mechanism of Action	Dose	Side Effects
D-penicillamine	Chelating action Cu water-soluble complex which increased urinary excretion Induction of melatonin Anti-inflammatory	Initial dose—150–300 mg/day. Gradual, weekly increase of the dose up to 20 mg/kg/day in 2 or 3 separate doses. Maintenance dose 10–20 mg/kg/day max., 750–1000 mg/day. A break between taking medication and meals is necessary. Important to remember the supplementation with pyridoxine. Treatment results appear after about 6 months.	Drug-induced lupus (joint pains, skin lesions and fewer) Proteinuria—damage to the glomeruli Myasthenia Swelling Joint pain Nausea Deterioration of neurological symptoms Bone marrow suppression–aplastic anaemia Hepatotoxicity
Zinc acetate/zinc sulphate	Reduces the absorption of copper—bioavailability. Induces the copper-binding metallothionein. Recommended for the following forms of the disease: neurological, asymptomatic or hepatic (slight increase in transaminases)	<6 years old 50 mg/day in 2 doses 6–16 years and the body weight of <50 kg 75 mg/day in 3 doses >16 years and the body weight of >50 kg 150 mg/day in 3 separate doses Minimum 1 h before meals and 2 h after meals.	Abdominal pain Nausea Gastritis/duodenitis Pancreatitis
Trientine	Chelating action	Initial dose 225 mg/day in 2–4 separate doses Maintenance treatment 12 years and under 225–750 mg/day in 2–4 doses 13 years and older 750–1250 mg/day Minimum 1 h before meals and 2 h after meals	Gastritis Rare aplastic anaemia and sideroblastic anaemia
Bis-choline tetrathiomolybdate	Increases biliary copper excretion Chelating action	20 mg 3× with meals	In observation Bone marrow depression Liver toxicity

**Table 6:** Prognostic scoring system in acute liver failure in the course of Wilson’s disease.

Score	Bilirubin (umol/L)	INR	AST (IU/L)	Leucocytosis	The Concentration of Albumins
0	0–100	0–1.29	0–100	0–6.7	>45
1	101–150	1.3–1.6	101–150	6.8–8.3	34–44
2	151–200	1.7–1.9	151–200	8.4–10.3	25–33
3	201–300	2.0–2.4	201–300	10.4–15.3	21–24
4	>300	>2.4	>300	>15.3	0–20

**Table 7:** Clinical symptoms of Menkes disease in relation to enzymatic disorders.

Affected Enzyme	Clinical Manifestations
Tyrosinase	Depigmentation of hair, pallor of skin.
Lysyl oxidase	Defect of elastine and collagen, split internal elastic layer of arteries. Vascular complication, bladder diverticula, osseous abnormalities.
Cytochrome-c-oxidase	Hypothermia, abnormal myelination. Myopathy, ataxia, seizures.
Ascorbate oxidase	Demineralization of bones.
Superoxide dismutase	Cytotoxic effects. Myelin degeneration. Spasticity, seizures.
Dopamineβ-hydroxylase	Abnormalities of catecholamines. Hypothalamic imbalances. Hypothermia, anorexia, somnolence, dehydration, respiratory failure, blood pressure decreased. Extrapyramidal symptoms. Ataxia.
Peptidylglycine—amidating monooxygenase	Reduced activity of melanocyte-stimulating hormone, corticotropin-releasing hormone, thyrotropin-releasing hormone, calcitonin, vasopressin.

**Table 8:** Neuropathological findings in the nervous system in Menkes disease.

Part of Nervous System	Abnormalities
Cerebral cortex	Severe loss of nerve cells accompanied by gliosis
Cerebral white matter	Gross deficiency of myelin and gliosis
Basal ganglia and thalamus	Mild loss of nerve cells. Sight hyperplasia of mitochondria.
Cerebellum	Severe atrophy of cortex with extensis gliosis. Decreased number of Purkinje cells—changes in size. Marked hyperplasia and hypertrophy of mitochondria with abnormal structure.
Brain stem	Myelination usually well presented
Spinal cord	Mild loss of nerve cells and slight gliosis
Peripheral nerves	Occasional degenerative changes
Eye	Retinal hypoplasia, atrophy of nerve fibres

**Table 9:** Comparison of Menkes and Wilson’s disease [37,39,50].

Parameter	Menkes Disease	Wilson’s Disease
Gene locus	Xq13.3	13q14.3
Inheritance	Sex-linked	Autosomal recessive
Impaired activity of copper-transporting ATP-ase	*ATP7A*	*ATP7B*
Defect	Intestinal copper absorption, deficiency of copper-containing enzymes	Biliary copper excretion, incorporation of copper into ceruloplasmin
Expression	All tissues except liver	Liver, to lesser extent kidneys, placenta Central nervous system
Age onset	Birth	Late childhood, adolescence
Symptomatic organs	Brain, hair, skin, genitourinary, gastrointestinal tracts, bone, eye	Liver, brain, cornea, RBC
Copper content in the body	Low	High
Concentration of free copper in plasma	Low	High
Concentration of copper in urine	Low	High
Ceruloplasmin	Decreased	Decreased
Treatment	Histidine–Copper injections supplementation Cupric chloride subcutaneously—non-effective	Zinc D—penicillamine Trientine

## Data Availability

No new data were created or analyzed in this study. Data sharing is not applicable to this article.
